# Mechanical Properties and Coagulation Characteristics of Flue Gas Desulfurization Gypsum-Based Polymer Materials

**DOI:** 10.3390/polym14214761

**Published:** 2022-11-06

**Authors:** Mingjing Li, Guodong Huang, Bo Wang, Yi Cui, Binbin Chang, Qiaoqiao Yin, Ming Ge, Shuwei Zhang, Qi Wang, Jiacheng Feng

**Affiliations:** 1 School of Civil Engineering and Construction, Anhui University of Science and Technology, Huainan 232001, China; 2 Engineering Research Center for Geological Environment and Underground Space of Jiangxi Province, East China University of Technology, Nanchang 330013, China; 3 Institute of Environment-Friendly Materials and Occupational Health, Anhui University of Science and Technology, Wuhu 241003, China; 4 School of Materials Science and Engineering, Tongji University, Shanghai 201800, China; 5 Wuhu Urban Construction Group Co., Ltd., Wuhu 241000, China; 6 Hefei Binhu Investment Holding Group Co., Ltd., Hefei 230091, China

**Keywords:** flue gas desulfurization gypsum, polymer, compressive strength, slag, condensation

## Abstract

To resolve problems caused by the accumulation of flue gas desulfurization gypsum (FGDG) in the environment, a polymer material was prepared using FGDG, granulated blast furnace slag (GBFS), fly ash (FA), and solid sodium silicate (SSS). The compressive strength of these polymer specimens cured for 3, 28, and 60 d was regularly measured, and their condensation behavior was analyzed. Both the formation behavior of mineral crystals and microstructure characteristics were analyzed further using X-ray diffraction and scanning electron microscopy. The compressive strength of pure FGDG polymer specimen (whose strength is generated by particle condensation crystallization) is insufficient and the condensation is slow. The addition of appropriate amounts of GBFS, FA, and SSS can continuously and considerably improve the compressive strength and shorten the setting time. The optimal proportions of FGDG, GBFS, and FA are 50%, 20%, and 30%, respectively, with the SSS addition amount of 20 g. The incorporation of GBFS, FA, and SSS can promote the polymerization of calcium, silicon, and aluminum in FGDG to form silicate and aluminosilicate minerals. Their formation is the main reason for the increased compressive strength and accelerated coagulation.

## 1. Introduction

Flue gas desulfurization gypsum (FGDG) is a type of industrial auxiliary gypsum obtained by desulfurization and purification of flue gas generated after the combustion of sulfur-containing fuel (e.g., coal) [1]. Its main component is SO_2_, which reacts with CaCO_3_ and generates calcium sulfate dihydrate (CaSO_4_·2H_2_O) through forced oxidation [2]. In the limestone gypsum flue gas desulfurization process, 2.7 ton of desulfurized gypsum can be generated for each 1 ton of SO_2_ removed [3]. A 300 MW coal-fired thermal power unit discharges about 30,000 ton of desulfurized gypsum every year (assuming 1.0% sulfur in coal) [4]. A substantial amount of desulfurized gypsum is generated as a by-product of limestone gypsum flue gas desulfurization systems. According to statistical data, in 2020, the desulfurized gypsum emissions of China reached about 147 million ton, which increased to more than 161 million ton in 2021 and continues increasing [5].

Gypsum can be used as a cement retarder or as a building material in products such as gypsum wallboard or gypsum ceiling [6]. However, because of technical problems associated with the desulfurization process, impurities, such as fly ash (FA), organic carbon, calcium carbonate, and soluble salts composed of sulfate or chloride of trace sodium, are inevitably mixed into FGDG. As a result, FGDG has a vastly different performance than natural gypsum, and therefore, it cannot be used as a retarder of cement or building decoration materials [7]. Moreover, large-scale FGDG landfill or its accumulation in the environment will not only damage soil and groundwater resources, but also pollute the air [8]. Therefore, research and development of recycling technologies specifically for FGDG have become increasingly important, with the goal of turning this waste into a valuable product while reducing its harm to the natural environment.

Caillahua et al. [9] used FGDG instead of natural gypsum as retarder of Portland cement. They found that FGDG retardation took 1 h longer compared to natural gypsum, and the composition of the mixture influenced its mechanical properties. Zhang et al. [10] prepared cementitious materials by mixing semi-dry flue gas desulfurization ash with hemihydrate gypsum and analyzed the microstructure of the product. The addition of desulfurized gypsum was found to increase the mixing water demand and prolong the setting time, while reducing the strength. The resulting product still met building requirements. Yang et al. [11] studied the effect of desulfurized gypsum on the properties of calcium sulfoaluminate cement. The results showed that FGDG can improve the hydration efficiency and promote the development of mechanical properties to a certain extent, but its content must not exceed 6%. Li et al. [12] showed that by adding desulfurized gypsum to concrete with a large amount of slag, the loss rate of early compressive strength of concrete was improved, the hydration reaction rate was accelerated, and shrinkage was alleviated. Liu et al. [13,14] used RM, FA, and DG as the main raw materials to prepare road base materials with different (CaO + Na_2_O)/(SiO_2_ + Al_2_O_3_) molar ratios, and investigated their mechanical properties, durability, microstructure and environmental performance.

The above studies have identified promising ways to utilize the resource of FGDG. However, most of them are limited to using FGDG either as retarder or mineral additive, the safe consumption of which is low, thus preventing its large-scale application [15]. FGDG is a calcium sulfate mineral with potential as a raw material for the preparation of polymer materials [16]. Compared with cement-based materials, polymer materials have superior mechanical properties and durability. They are also low carbon, energy conserving, and environmentally benign [17,18]. In this study, FGDG was used as the main raw material to prepare a high-content and high-performance FGDG-based polymer material. The effects of various proportions of raw materials on compressive strength and coagulation behavior were analyzed in depth. The characteristics of the mineral crystal structure of this polymer material were analyzed by X-ray diffraction (XRD), and the characteristics of both the microstructure and polymerization reaction between micro particles were analyzed by scanning electron microscopy (SEM).

## 2. Experimental Materials and Methods

### 2.1. Experimental Materials

#### 2.1.1. Raw Materials

The utilized FGDG was produced by Huaihu Coal and Power Co., Ltd. (Huainan, China) through the limestone gypsum wet flue gas desulfurization process. Prior to experiments, FGDG was dried for 3 h at 60 °C. The specific surface area of FGDG was 302 m^2^/kg. Less than 36% of the particle size range reached the margin of a square hole sieve (45 μm). The chemical composition of FGDG (obtained by X-ray fluorescence (XRF) spectrum analysis) is shown in Table 1. The main components are calcium and sulfur, accounting for 34.42% and 38.21% of the total, respectively, while the contents of silicon and aluminum only account for 3.24% and 1.57%, respectively. FGDG is a raw material with high calcium and sulfur contents and low silicon and aluminum contents. However, the loss on ignition is as high as 19.78%, indicating that FGDG still contains a large amount of free and bound water.

S95 grade granulated blast furnace slag (GBFS) was obtained from Shandong Kangjing New Material Technology Co., Ltd. (Dezhou, China). The specific surface area of GBFS reached 406 m^2^/kg, and less than 12% of the particle fineness reached the margin of a square hole sieve (45 μm). Its performance conforms to GBFS used in cement and concrete (GB/T 18046-2017) [19]. The chemical composition of GBFS is shown in Table 1. Compared with FGDG, the contents of calcium (34.23%), silicon (34.65%), and aluminum (20.72%) in GBFS are more balanced.

Class F (low calcium ash) grade I FA was also obtained from Shandong Kangjing New Material Technology Co., Ltd. (Dezhou, China). The specific surface area of FA was 411 m^2^/kg, and less than 12% of the particle fineness reached the margin of a square hole sieve (45 μm). The chemical composition of FA is shown in Table 1. The main components are silicon and aluminum, accounting for 53.89% and 31.24% of the total, respectively, while the content of calcium only accounts for 2.18%.

#### 2.1.2. Alkali Activator

Sodium hydroxide and solid sodium silicate (SSS; Na_2_SiO_3_·9H_2_O) were used as alkali activators, both of which were obtained from Tianjin Jindong Tianzheng Fine Chemical Reagent Factory (Tianjing, China). Both were of analytical purity, with the purity exceeding 99.5%.

#### 2.1.3. Others

The sand was medium coarse river sand (after cleaning and drying), with a fineness modulus of 2.8 in the dry state. Tap water was used for the test.

### 2.2. Experimental Methods

#### 2.2.1. Experimental Mix Proportion

Polymer specimen L-1 was prepared entirely from FGDG and was used to test the mechanical properties and coagulation characteristics of FGDG. From polymer specimens L-2 to L-6, the content of GBFS was increased in intervals of 10%, and the content of FGDG was reduced accordingly (i.e., by 10% for each 10% GBFS addition), to study the reinforcement and modification effects of GBFS on FGDG. In polymer specimens L-7–L-11, the GBFS content remained unchanged, and the FA content was gradually increased (by intervals of 10%). The FGDG content was reduced accordingly (i.e., by 10% for each 10% of FA addition), to study the modification effect of the increased silicon level in FA on FGDG. From polymer specimens L-12 to L-15, the doping amount of FGDG, GBFS, and FA were left unchanged, while the doping amount of SSS was gradually increased (by intervals of 10%) to study the silicon increasing excitation effect of SSS. The data before (“/”) in Table 2 show the mix proportion of each group of mortar specimens for compressive strength tests, while the data after (“/”) show the mix proportion of paste specimens for coagulation behavior tests.

#### 2.2.2. Preparation and Curing of Specimens

Laboratory preparation methods of FGDG polymer specimens were employed according to the standard of Testing method of cements—Determination of strength (ISO method, GB/T 17671-2021) [20]. The preparation method of polymer specimen L-12 (mortar, used for compressive strength test) is explained in detail as an example. First, sodium hydroxide was dissolved in water and left to cool. Then, FGDG, GBFS, FA, and SSS were mixed (W-20 V-type mixer, Changzhou Yimai Machinery Manufacturing Co., Ltd., Jiangsu, China) for 2 min according to the mix proportion in Table 2. This step ensured that different raw materials were mixed evenly. Then, the mixture was placed into a blender (NJ-160A cement paste blender, Hebei Xingji Instrument Equipment Co., Ltd., Shijiazhuang, Hebei, China), sodium hydroxide solution was added, and slow mixing was continued for 30 sec. Then, test sand was added, and the mixture was rapidly stirred for 60 sec. Finally, the mixture was poured into a cuboid mold with a size of 40 mm × 40 mm × 160 mm. The preparation of paste specimens (to study condensation behavior) was carried out based on the mortar specimen L-12, omitting the addition of test sand.

Immediately after preparation, mortar specimens were placed in a curing room, and the formwork was removed after 3 d of curing. After formwork removal, specimens remained in the curing room for curing until the experimental design age was reached. The temperature was maintained at 20 ± 2 °C and the humidity exceeded 98%. Coagulation behavior tests were conducted immediately following the preparation of paste samples. The laboratory temperature was 26 °C, and the humidity was 60%.

#### 2.2.3. Macroscopic Experiments

(1) Compressive strength test

A DYE-2000 press (Jinan Zhongchuang Industrial Test System Co., Ltd., Jinan, Shandong, China) was used to test the compressive strength of specimens cured for 3 d, 28 d, and 60 d, and to analyze the influence of different proportions on the development of compressive strength (loading speed: 2.4 ± 0.2 kN/s). The arithmetic mean of six data points was taken as the experimental result.

(2) Condensation behavior test

The condensation behavior experiment was carried out using the standard Vicat instrument (Xingjian Instrument Equipment Co., Ltd., Cangzhou, Hebei, China) in reference to the Test methods for water requirement of normal consistency. Time and soundness settings of Portland cement (GB/T 1346-2011) [21] were used to deeply analyze the influence of raw materials on the coagulation characteristics.

#### 2.2.4. Microscopic Experiments

(1) X-ray diffraction analysis

A smart Lab SE intelligent X-ray diffractometer (RIGAKU, Tokyo, Japan) was used to analyze the mineral crystal composition of specimens (after curing for 28 d). The influence of the formation and transformation of mineral crystals on the development of compressive strength and condensation behavior was analyzed.

(2) Scanning electron microscopy analysis

The microstructure of specimens was analyzed by a Hitachi flexSEM 1000 scanning electron microscope (Hitachi, Tokyo, Japan). The bonding characteristics of micro particles, the structural characteristics of polymerization products, and the effects of different raw materials on the microstructure were observed under 1000× magnification.

## 3. Experimental Results and Discussion

### 3.1. Compressive Strength Analysis

#### 3.1.1. Compressive Strength of Flue Gas Desulfurization Gypsum Polymer Specimens

**Figure 1:**
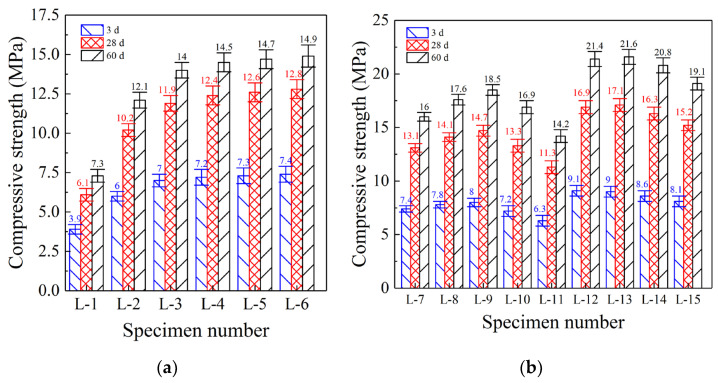
Compressive strength analysis. (**a**) Specimens L-1 to L-6. (**b**) Specimens L-7 to L-15.

Figure 1 and Table 3 shows the compressive strength development of FGDG polymer mortar specimens at different curing ages. Under a liquid-solid ratio of 0.5 and excitation of sodium hydroxide solution, the compressive strength of specimen L-1 (Figure 1a; 100% FGDG) was clearly insufficient, at only 3.9 and 6.1 MPa after curing for 3 d and 28 d, respectively. Even at a curing age of 60 d, the compressive strength only reached 7.3 MPa. Specimen L-1 (i.e., FGDG) can only be used as a non-load bearing filling material, which lacks market application prospects. The reason for this is that the compressive strength development of polymer specimens mainly depends on the formation of silicate and aluminosilicate minerals [22]. However, their formation not only requires calcium, but also large amounts of silicon and aluminum. XRF analysis showed that although the high calcium content of FGDG (34.42%, Table 1) was favorable for the formation of polymerization products, the low contents of silicon (3.24%) and aluminum (1.57%) seriously hindered their formation, which affected the compressive strength development. Therefore, the compressive strength of FGDG has to be considerably enhanced by adding silicon and aluminum so it can be used as load-bearing structural material, thus expanding its market application range.

#### 3.1.2. Enhancing the Compressive Strength of Granulated Blast Furnace Slag

The compressive strength of specimen L-2 (Figure 1a; 90% FGDG and 10% GBFS) reached 6.0 MPa (3 d), 10.2 MPa (28 d), and 12.1 MPa (60 d), which are 53.8%, 67.2%, and 65.8% higher, respectively, than the compressive strength of specimen L-1 (100% FGDG). GBFS contains more balanced amounts of silicon (34.65%; Table 1) and aluminum (20.72%), which compensated for the defect of insufficient silicon and aluminum contents in specimen L-1. Under strong alkaline excitation conditions established by sodium hydroxide, both silicon and aluminum (dissolved from GBFS) polymerize with calcium (dissolved from FGDG) to form silicate and aluminosilicate minerals [23]. Their formation markedly improved the compressive strength of specimen L-2, as further demonstrated in the XRD analysis shown in Section 3.3.2. Moreover, the compressive strength of specimen L-3 (Figure 1a; 80% FGDG and 20% GBFS) was also considerably improved. Strengths of specimen L-3 reached 17.2% (3 d), 16.8% (28 d), and 15.9% (60 d), exceeding those of specimen L-2 (90% FGDG and 10% GBFS), but the increasing range was reduced to a certain extent. With gradual increase in GBFS content, the proportion of silicon and aluminum in specimen L-3 also gradually increased. This improved the polymerization efficiency and prevented the inability of active calcium (from FGDG) to polymerize because of the insufficient content of silicon and aluminum in FGDG [24].

However, the compressive strength of specimen L-4 (Figure 1a; 70% FGDG and 30% GBFS) increased further, but only by 2.9% (3 d), 3.8% (28 d), and 3.5% (60 d) compared to that of specimen L-3 (80% FGDG and 20% GBFS). The strength growth rate began to decrease noticeably. Moreover, the compressive strengths of specimens L-5 (60% FGDG and 40% GBFS) and L-6 (50% FGDG and 50% GBFS) did not continue to increase compared with that of specimen L-4. Increasing the content of GBFS beyond 20% did not increase the utilization of silicon and aluminum contents. Although the contents of silicon and aluminum in GBFS were notably higher than in FGDG (Table 1), the content of calcium in GBFS (34.65%) was basically the same as that in FGDG (34.42%). With increase in GBFS content, the calcium content also increased in the same proportion. As a result, specimens L-5 and L-6 still lacked silicon and aluminum. The formation of silicate and aluminosilicate cannot be considerably increased even if the GBFS content is continuously increased beyond 20%, which explains why the compressive strengths of specimens L-5 to L-6 did not increase further [25]. Therefore, the best GBFS content was 20% and silicon and aluminum contents were further increased by adding FA, as described in the following sections.

#### 3.1.3. Enhancing Compressive Strength by Adding Fly Ash

The compressive strength of specimen L-7 (Figure 1b; 70% FGDG, 20% GBFS, and 10% FA) continued to grow, and strengths were 6.1% (3 d), 10.2% (28 d), and 14.5% (60 d) higher compared to specimen L-3 (80% FGDG and 20% GBFS). The addition of FA continued to improve the compressive strength, and the increasing range in later curing periods (28 d and 60 d) was much higher than that in the early period (3 d). FA contains large amounts of silicon (53.89%; Table 1) and aluminum (31.24%), which are considerably higher than their amounts in GBFS (34.65% and 20.72%). Therefore, the efficiency of increasing silicon and aluminum of FA exceeds that of GBFS. More importantly, the calcium content in FA was only 2.18%. Despite its ability to greatly increase the contents of silicon and aluminum, this did not lead to further increase in the calcium content. Thus, the emergence of a large amount of calcium residue was avoided, and the compressive strength of the specimen was further improved. The compressive strength of specimen L-8 (Figure 1b; 60% FGDG, 20% GBFS, and 20% FA) grew continuously. Moreover, the compressive strength of specimen L-9 (Figure 1b; 50% FGDG, 20% GBFS, and 30% FA) was still more than 5% higher than that of specimen L-8. Continuously increasing the FA content continuously optimized the proportions of calcium, silicon, and aluminum in specimens L-8 and L-9 and promoted the formation of high-strength silicate and aluminosilicate, thus continuously promoting the increase in compressive strength.

However, the compressive strength of specimen L-10 (Figure 1b; 40% FGDG, 20% GBFS, and 40% FA) decreased, and was 10.4% (3 d), 9.8% (28 d), and 8.8% (60 d) lower than the compressive strength of specimen L-9 (50% FGDG, 20% GBFS, and 30% FA). Moreover, the compressive strength of specimen L-11 (Figure 1b; 30% FGDG, 20% GBFS, and 50% FA) further decreased by more than 15% (compared with specimen L-10). The compressive strength decreased with increase in FA content (exceeding 30%), and the decreasing range increased continuously. Continuous increase in the FA content inevitably led to decrease in FGDG and GBFS contents, which caused a continuous increase in silicon and aluminum contents and a significant decrease in calcium content. To form high-strength silicates or aluminosilicates, silicon and aluminum must be polymerized with calcium. If the calcium content is insufficient, silicon and aluminum will be available in excess and cannot be polymerized, thus leading to a reduction of compressive strength [26]. Therefore, the best enhancement effect was achieved at FA content of 30%.

#### 3.1.4. Enhancing Compressive Strength by Adding Solid Sodium Silicate

The compressive strength of specimen L-12 (Figure 1b; 50% FGDG, 20% GBFS, 30% FA, and 20 g SSS) grew continuously, and was 13.5% (3 d), 15.2% (28 d), and 15.8% (60 d) higher than that of specimen L-9 (50% FGDG, 20% GBFS, and 30% FA). SSS addition can further improve the compressive strength by increasing the silicon content. However, compared with specimens L-10 and L-11, the silicon of FA led to a decrease in compressive strength. The reason for this is the essentially different silicon increasing effect of SSS compared to that of FA. First, the silicon increasing effect of SSS does not lead to a decrease in the calcium content as is the case with FA [27]. Second, SSS releases silicon by dissolution. The silicon in FA must undergo depolymerization under strong alkali excitation, and then dissolve into the reaction solution. However, the silicon in SSS can only directly increase silicon by dissolution [28]. Therefore, judging from the increase in efficiency and adverse effects (resulting in a decrease in calcium content), SSS is clearly superior to FA. Therefore, the compressive strength of specimen L-12 can still be increased.

However, the compressive strength of specimen L-13 (Figure 1b; 50% FGDG, 20% GBFS, 30% FA, and 40 g SSS) did not increase further with higher SSS contents. Instead, the compressive strengths of specimens L-14 (Figure 1b; 50% FGDG, 20% GBFS, 30% FA, and 60 g SSS) and L-15 (Figure 1b; 50% FGDG, 20% GBFS, 30% FA, and 80 g SSS) decreased considerably. Since a liquid-solid ratio of 0.5 was maintained, the solubility of SSS remained unchanged, and the SSS content was continuously increased. When the maximum SSS solubility was exceeded, residual SSS was inevitably formed. However, this surplus SSS cannot continue to increase the silicon content. Instead, its crystal structure affects the compactness of the microstructure, resulting in a reduction of compressive strength [29]. Therefore, the optimal amount of SSS was determined to be 20 g.

### 3.2. Condensation Behavior Analysis

#### 3.2.1. Condensation Behavior of the Flue Gas Desulfurization Gypsum Specimen

**Figure 2:**
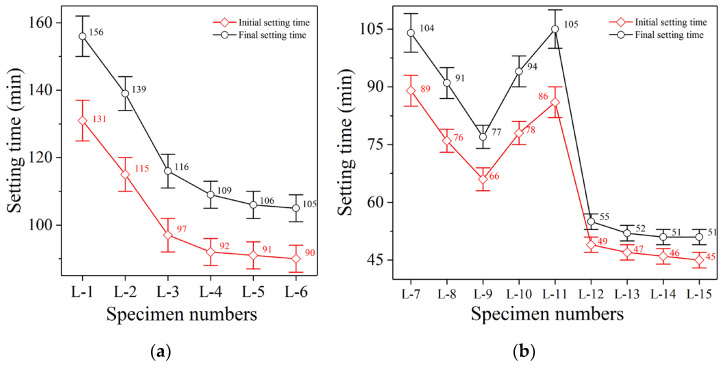
Condensation behavior analysis. (**a**) Specimens L-1 to L-6. (**b**) Specimens L-7 to L-15.

The setting behavior of polymer specimen L-1 (100% FGDG) is shown in Figure 2a. The initial setting time (IST) was 131 min, while the final setting time (FST) was 156 min. The longer IST is beneficial to the application of FGDG polymer materials because of long-distance transportation from the mixing plant (which is often required) as well as needs imposed by pouring and construction [30]. When FGDG meets the reaction solution, it can form a protective film on its surface to block the dissolution of active substances from FGDG. Thus, the polymerization reaction of FGDG particles was effectively delayed and the reaction was retarded.

#### 3.2.2. Effect of Granulated Blast Furnace Slag on Condensation Behavior

The IST and FST of polymer specimen L-2 (Figure 2a; 90% FGDG and 10% GBFS) shortened to 115 min and 139 min, which are 12.2% and 10.9% lower than those of specimen L-1 (100% FGDG), respectively. When GBFS was not added, specimen L-1 could only dissolve and precipitate colloidal particles through FGDG. As the FGDG slurry continued to thicken, colloidal particles gradually agglomerated into crystals. These crystals gradually grew, then coexisted and interlaced with each other. Ultimately, this led to a continuous increase in the compressive strength of the FGDG slurry [31]. However, with the addition of 10% GBFS, specimen L-2 not only coagulated through the crystallization of FGDG particles, but was also polymerized by silicon and aluminum dissolved from GBFS and calcium from FGDG. Consequently, silicate and aluminosilicate minerals formed, thus accelerating the coagulation of specimen L-2.

The IST and FST of polymer specimen L-3 (Figure 2a; 80% FGDG and 20% GBFS) further shortened to 97 min and 116 min, which are 15.7% and 16.5% lower than those of specimen L-2 (90% FGDG and 10% GBFS), respectively. With increase in GBFS content, the amount of active silicon and aluminum increased strongly, while the amount of active calcium provided by FGDG was still in a state of surplus. This active calcium finally accelerated the formation of silicate and aluminosilicate minerals, thus accelerating the coagulation of specimen L-3.

However, the IST and FST of polymer specimen L-4 (Figure 2a; 70% FGDG and 30% GBFS) shortened to 92 min and 109 min, which are only 5.2% and 6.0% lower than those of specimen L-3 (80% FGDG and 20% GBFS), respectively. When the content of GBFS was increased to 30%, the shortening range was noticeably reduced. Moreover, the setting time of specimens L-5 (Figure 2a; 60% FGDG and 40% GBFS) and L-6 (Figure 2a; 50% FGDG and 50% GBFS) almost had the same shortening range as that of specimen L-4. GBFS contains a large amount of calcium, not less than FGDG. Increasing the GBFS content will still result in a large calcium surplus. Therefore, when the content of GBFS exceeds 30%, the surplus calcium cannot further accelerate the polymerization reaction rate and promote the formation of polymerization products [32], which cannot further accelerate the setting time.

#### 3.2.3. Effect of Fly Ash on Condensation Behavior

The IST and FST of polymer specimen L-7 (Figure 2b; 70% FGDG, 20% GBFS, and 10% FA) further shortened to 89 min and 104 min, which are 8.2% and 10.3% lower than those of specimen L-3 (80% FGDG and 20% GBFS), respectively. If increasing the amount of GBFS cannot continue to accelerate coagulation and improve compressive strength, the addition of FA can further accelerate the coagulation and continue to improve compressive strength. This does not mean that the polymerization activity of FA is higher than that of GBFS, but the proportion of calcium, silicon, and aluminum can be further optimized by reducing the FGDG content and increasing the FA content. Moreover, with increase in FA content, the setting time of specimens L-8 (Figure 2b; 60% FGDG, 20% GBFS, and 20% FA) and L-9 (Figure 2b; 50% FGDG, 20% GBFS, and 30% FA) showed a trend of continuous shortening. This indicates that increasing the FA content and decreasing the FGDG content can continue to optimize the ratio of calcium, silicon, and aluminum [33].

However, the IST and FST of polymer specimen L-10 (Figure 2b; 40% FGDG, 20% GBFS, and 40% FA) increased to 78 min and 94 min, which are 18.2% and 22.1% higher than those of specimen L-9, respectively. Moreover, the IST and FST of specimen L-11 (Figure 2b; 30% FGDG, 20% GBFS, and 50% FA) further increased to 86 min and 105 min, respectively. The condensation behavior indicates that when the FA content exceeds 30%, the coagulation cannot be further accelerated, and the coagulation time is extended. The proportion of calcium, silicon, and aluminum in specimens L-10 and L-11 gradually progressed in an unfavorable way. The FGDG content was continuously reduced while the FA content was continuously increased, which led to excess silicon and aluminum but not enough calcium. This caused a slowdown of the polymerization rate and a decreased formation rate of silicate and aluminosilicate minerals [34]. Therefore, specimen L-9 (50% FGDG; 20% GBFS and 30% FA) had the best mixing ratio, as it possessed both high compressive strength and a suitable setting time.

#### 3.2.4. Effect of Solid Sodium Silicate on Condensation Behavior

The IST and FST of polymer specimen L-12 (Figure 2b; 50% FGDG, 20% GBFS, 30% FA, and 20 g SSS) shortened to 49 min and 55 min, which are 25.8% and 28.6% higher than those of specimen L-9 (Figure 2b; 50% FGDG, 20% GBFS, and 30% FA), respectively. Addition of SSS considerably accelerated the coagulation of specimen L-12, which further improved its polymerization rate and accelerated the formation of polymerization products by dissolving and releasing sodium and silicon [35]. However, FA also increased the silicon content, and addition of more than 30% FA prolonged coagulation. The reason for this result is that the silicon increasing mechanism of SSS is completely different from that of FA. SSS releases silicon into the reaction solution through dissolution, which can achieve the goal of increasing the silicon content without changing the ratio of calcium, silicon, and aluminum in specimen L-12. However, when the amount of FA was increased to increase silicon and aluminum contents, it inevitably led to a decrease in the FGDG content, and in contrast, adversely affected the decrease in calcium content [36]. Increasing the silicon content by increasing the FA content also inevitably introduced a large volume of inert particles (inherent in FA). These inert particles have no reaction activity, only play the role of fillers, and cannot play a favorable role in condensation and development of compressive strength [37]. However, inert particles are not produced in the dissolution of SSS. Therefore, condensation and the development of compressive strength are not adversely affected.

The IST and FST of polymer specimen L-13 (Figure 2b; 50% FGDG, 20% GBFS, 30% FA, and 40 g SSS) shortened to 47 min and 52 min, which are 4.1% and 5.5% lower than those of specimen L-12, respectively. Moreover, the setting time of specimens L-14 (Figure 2b; 50% FGDG, 20% GBFS, 30% FA, and 60 g SSS) and L-15 (Figure 2b; 50% FGDG, 20% GBFS, 30% FA, and 80 g SSS) did not shorten further. The reason is that once the SSS content exceeds maximum solubility, SSS solids are deposited. The remaining SSS cannot continue to increase the silicon content, but instead becomes inert particles that fill the specimen [38]. Therefore, when the content of SSS is too high (i.e., exceeding 20 g), SSS not only promotes the polymerization reaction, but also undermines the development of compressive strength. Therefore, the optimal SSS content was determined to be 20 g.

### 3.3. X-ray Diffraction Analysis

#### 3.3.1. X-ray Diffraction Analysis of Flue Gas Desulfurization Gypsum Specimens

**Figure 3:**
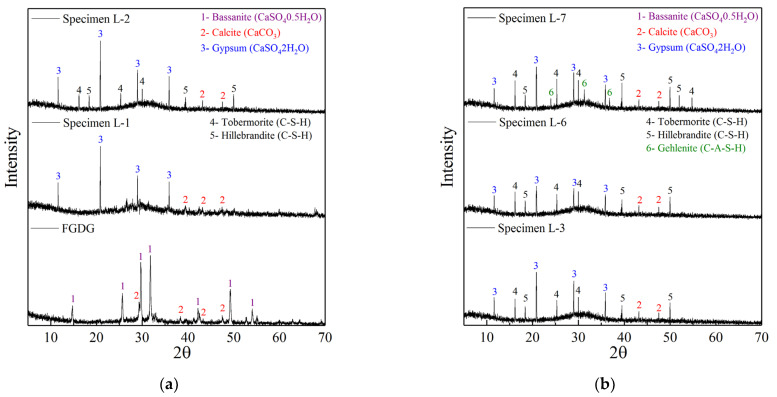
XRD analysis. (**a**) Specimens L-1 to L-2. (**b**) Specimens L-3 to L-7.

XRD analysis of the FGDG raw material (Figure 3a) showed that FGDG was mainly composed of bassanite mineral (CaSO_4_·0.5H_2_O) with a small amount of calcite crystals (CaCO_3_). However, in specimen L-1, which was completely prepared by FGDG, mineral crystals were completely converted from bassanite to gypsum (CaSO_4_·2H_2_O). Moreover, no crystals of silicate and aluminosilicate minerals appeared in specimen L-1, and the characteristic peak of calcite did not increase noticeably. Specimen L-1 was completely derived from the hydration reaction of bassanite, which produces gypsum. Bassanite first dissolves in the reaction solution, then quickly forms a saturated solution and generates gypsum. As the solubility of gypsum is much lower than that of bassanite (only one fifth of the solubility of bassanite), gypsum continuously precipitates and crystallizes [39]. The crystals grow gradually, and then coexist and interlace with each other. Consequently, specimen L-1 condensed and established strength.

#### 3.3.2. X-ray Diffraction Analysis of Granulated Blast Furnace Slag Enhanced Specimens

The XRD pattern of polymer specimen L-2 (Figure 3a; 90% FGDG and 10% GBFS) showed characteristic peaks of gypsum, indicating that gypsum still precipitated and crystallized, thus promoting the condensation and the development of compressive strength. The most important difference between specimens L-2 and L-1 was the emergence of characteristic peaks of tobermorite (Ca_5_Si_6_O_16_(OH)_2_·4H_2_O) and hillebrandite (Ca_2_SiO_3_(OH)_2_), which appeared in specimen L-2 but not in specimen L-1. GBFS provides a large amount of active silicon and aluminum. Therefore, the active calcium in FGDG can polymerize with silicon and aluminum to form silicate minerals (tobermorite and hillebrandite) [40]. These are key factors for accelerating the solidification and improving the compressive strength of specimen L-2.

The mineral crystal structure of polymer specimen L-3 (Figure 3b; 80% FGDG and 20% GBFS) was further optimized. Compared with specimen L-2 (90% FGDG and 10% GBFS), the intensities of the characteristic peaks of tobermorite and hillebrandite increased considerably in specimen L-3, while the intensity of the characteristic peaks of gypsum decreased. With increase in GBFS content, the amount of silicon and aluminum increased markedly. Consequently, more calcium (from FGDG) polymerized with silicon and aluminum, resulting in a decrease in amount of gypsum crystals, which is the reason for the decrease in the characteristic gypsum peak. Compared with the condensation, crystallization, and precipitation of gypsum, the formation of silicate has a more apparent effect on promoting the condensation and development of compressive strength [41]. Therefore, the condensation of specimen L-3 was further accelerated, and the compressive strength was further developed.

The characteristic peaks of tobermorite and hillebrandite in polymer specimen L-6 (Figure 3b; 50% FGDG and 50% GBFS) were not considerably higher than those in specimen L-3. This indicates that increasing the GBFS content did not greatly promote the formation of silicate minerals, and explains why the setting time of specimen L-6 did not shorten and the compressive strength did not increase considerably. However, the strength of the characteristic gypsum peak in specimen L-6 clearly decreased compared with that of specimen L-3. The increase in GBFS content led to a decrease in FGDG content, resulting in a significant decrease in the amount of bassanite, and thus a significant reduction of the amount of gypsum formed by hydration of bassanite. This explains why the characteristic peak of gypsum in specimen L-6 was clearly lower than that of specimen L-3.

#### 3.3.3. X-ray Diffraction Analysis of Fly Ash Enhanced Specimens

Incorporation of FA further increased the characteristic peak of polymer specimen L-7 (Figure 3b; 70% FGDG, 20% GBFS, and 10% FA). The intensities of the characteristic peaks of tobermorite and hillebrandite were much higher in specimen L-7 than in specimen L-3, and new characteristic peaks of tobermorite and hillebrandite appeared in specimen L-7 that did not appear in specimen L-3. These phenomena indicate that addition of FA further promoted the formation of silicate minerals. Most importantly, the characteristic peak of gehlenite (2CaO·Al_2_O_3_·SiO_2_) also appeared in specimen L-7, which did not appear in specimen L-3. Gehlenite, which is a kind of aluminosilicate mineral, is formed by the polymerization of calcium, silicon, and aluminum, and the incorporation of FA is the main reason for its formation [42]. Therefore, addition of FA increased the formation of silicate and aluminosilicate minerals, which explains the further acceleration of coagulation and the better development of compressive strength.

**Figure 4:**
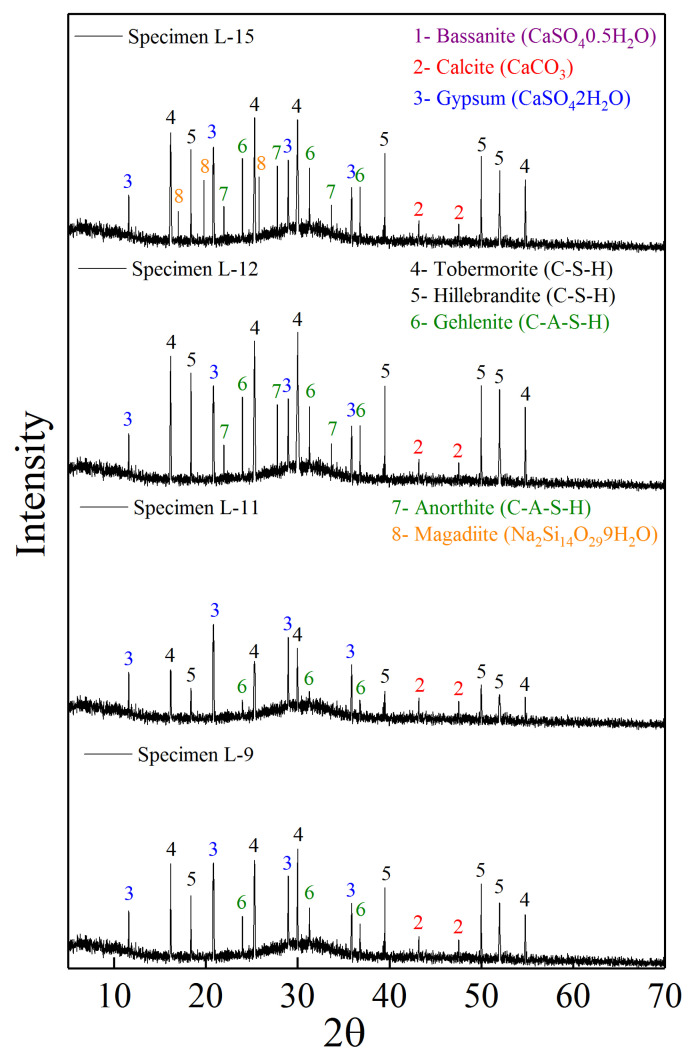
XRD analysis of Specimens L-9 to L-15.

Compared with specimen L-7, the characteristic peak intensities of tobermorite and hillebrandite in polymer specimen L-9 (Figure 3b; 50% FGDG, 20% GBFS, and 30% FA) still increased considerably, and the characteristic peak of gehlenite also increased. Increasing the amount of FA can continuously increase silicon and aluminum, so that the excess calcium in GBFS and FGDG can fully polymerize and form silicate and aluminosilicate minerals. This formation of aluminosilicate minerals continuously accelerated the setting time of specimen L-9 and improved its compressive strength. However, the characteristic peaks of tobermorite and hillebrandite in specimen L-11 (Figure 4; 30% FGDG, 20% GBFS, and 50% FA) were markedly lower, and the intensity of the characteristic peaks of gehlenite was also considerably reduced. If the FA content exceeds 30%, the polymerization reaction of calcium, silicon, and aluminum is seriously affected, and the formation of silicate and aluminosilicate minerals is reduced [43]. Thus, the setting time of specimen L-11 was prolonged, and its compressive strength decreased.

#### 3.3.4. X-ray Diffraction Analysis of Solid Sodium Silicate Enhanced Specimens

The mineral crystal characteristics of polymer specimen L-12 (Figure 4; 50% FGDG, 20% GBFS, 30% FA, and 20 g SSS) were notably improved compared with specimen L-9 (Figure 3b; 50% FGDG, 20% GBFS, and 30% FA). The characteristic peak intensities of tobermorite and hillebrandite increased further, and the characteristic peak intensity of gehlenite also increased further. Moreover, the characteristic peak of anorthite (CaO·Al_2_O_3_·2SiO_2_) appeared in specimen L-9, which did not appear in previously analyzed specimens. Anorthite is an important aluminosilicate mineral, and its formation greatly impacts the coagulation of accelerated time and the improvement of compressive strength [44]. Therefore, the increase in intensity of characteristic peaks of tobermorite, hillebrandite, and gehlenite, as well as the appearance of characteristic peaks of anorthite, indicated that the polymerization reaction efficiency was further improved. This further accelerated coagulation and continuously increased compressive strength.

However, the characteristic peaks of tobermorite, hillebrandite, gehlenite, and anorthite in polymer specimen L-15 (Figure 4; 50% FGDG, 20% GBFS, 30% FA, and 80 g SSS) did not increase with increase in SSS content compared with specimen L-12 (Figure 4; 50% FGDG, 20% GBFS, 30% FA, and 20 g SSS). Increasing the SSS content did not promote the formation of silicate and aluminosilicate minerals. Moreover, characteristic magadiite (Na_2_Si_14_O_29_·9H_2_O) peaks appeared in specimen L-15. Magadiite is a sodium silicate mineral, and its emergence indicates that increasing the SSS content does not increase the content of silicon at all. Instead, insoluble SSS self-condenses to form magadiite which then precipitates in specimen L-15 [45]. The strength of magadiite precipitation is far lower than that of silicate and aluminosilicate, and its formation is the reason for the decrease in compressive strength of specimen L-15.

### 3.4. Scanning Electron Microscopic Analysis

#### 3.4.1. Microstructure of Polymer Specimen L-1

**Figure 5:**
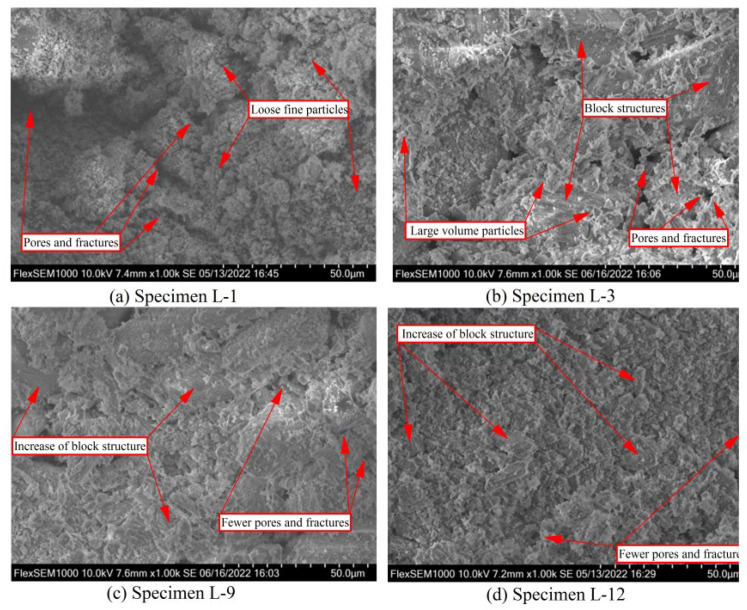
SEM analysis of specimens L-1 to L-12.

The microstructure of polymer specimen L-1 (Figure 5a; 100% FGDG) was completely composed of fine particles, and no large blocks or gel structures were formed. Particles were loosely arranged, and the adhesive force was clearly insufficient. There was no polymerization reaction of FGDG particles, and particles were bound together by condensation crystallization. Moreover, many pores, cracks, and other defects were seen on the surface, indicating that the reaction degree of crystal condensation was low, which is also the reason for the clearly insufficient compressive strength of specimen L-1.

#### 3.4.2. Microstructure of Polymer Specimen L-3

The incorporation of GBFS clearly improved the microstructure. Compared with specimen L-1, many block structures appeared in polymer specimen L-3 (Figure 5b; 80% FGDG and 20% GBFS). Moreover, although granular structures were still present in specimen L-3, the volume of particles was considerably higher than that in specimen L-1, and the bonding strength between particles was also much higher. After incorporation of GBFS, the original condensed crystals form of FGDG changed to the structural form of silicate and aluminosilicate formed by the polymerization of active calcium, silicon, and aluminum, leading to the formation of the large granular structure [46]. The formation of substantial amounts of silicate and aluminosilicate minerals accelerated the setting time and promoted the compressive strength growth of specimen L-3. However, many defects, such as pores and cracks, persisted in specimen L-3, indicating that the degree of polymerization reaction was still insufficient.

#### 3.4.3. Microstructure of Polymer Specimen L-9

Addition of FA further improved the microstructure of polymer specimen L-9 (Figure 5c; 50% FGDG, 20% GBFS, and 30% FA). Compared with specimen L-3, the block structure was more apparent in specimen L-9, and the integrity was better. Fewer large granular substances can be found, indicating that more granular substances participated in the polymerization reaction to form massive silicate and aluminosilicate minerals. Moreover, although defects, such as pores and cracks, still persisted in specimen L-9, the number and size of pores and cracks were considerably lower than those in specimen L-3. Therefore, addition of FA further enhanced the polymerization reaction and notably improved the microstructure, thus further accelerating the coagulation and greatly improving the compressive strength [47].

#### 3.4.4. Microstructure of Polymer Specimen L-12

Compared with specimen L-9, the microstructure of polymer specimen L-12 (Figure 5c; 50% FGDG, 20% GBFS, 30% FA, and 20 g SSS) was more compact and its integrity was better. Scattered microparticles were barely observed on the surface of specimen L-12, and massive silicate and aluminosilicate minerals were formed. Moreover, specimen L-12 exhibited considerably fewer pores and cracks. Therefore, the addition of an appropriate amount of SSS further improved the microstructure, promoted the formation of polymerization products, and improved the polymerization reaction efficiency [48]. Overall, this further accelerated the coagulation of specimen L-12 and markedly improved its compressive strength.

## 4. Conclusions

In this study, FGDG polymer specimens were prepared by different mix proportions. XRF was used to determine the content of various elements in FGDG, GBFS, and FA. The influence of different proportions of these elements on the development of compressive strength and condensation behavior was analyzed. Moreover, the effects of GBFS, FA, and SSS on the formation of mineral crystals were further analyzed by XRD. Finally, the microstructure and morphological characteristics were analyzed by SEM. The results are summarized as follows:

(1) The compressive strength of the pure FGDG specimen is clearly insufficient (only 6.1 MPa after curing for 28 d), and it needs a long time to set. If FGDG is not modified and reinforced, it cannot be used as structural load-bearing material. Therefore, the goal of turning waste into a valuable resource cannot be realized.
(2) Optimal amounts of GBFS, FA, and SSS can continuously and considerably increase the compressive strength and accelerate setting, even beyond that of cement-based materials with the same curing age. When 50%, 20%, and 30% of FGDG, GBFS, and FA were added, respectively, and 20 g of SSS was also added, the obtained mechanical properties were the best among all tested specimens. Specifically, the increase ranges were 95.1%, 23.5% and 15%, respectively and GBFS had the most obvious improvement effect. However, the excessive incorporation of FGDG, GBFS, FA, and SSS resulted in a decrease in compressive strength and an extension of the setting time.
(3) XRD analysis showed that the pure FGDG specimen could not polymerize and form silicate and aluminosilicate minerals, which is the reason for the insufficient compressive strength and slow setting of this specimen. However, the addition of appropriate amounts of GBFS, FA, and SSS increased calcium and silicon contents stepwise, thus promoting the formation of different types of silicates and aluminosilicate minerals. Overall, this greatly improved the compressive strength and accelerated the setting of the specimen.
(4) SEM analysis showed that the strength of the pure FGDG specimen was produced by the condensation and crystallization of FGDG particles. However, the incorporation of GBFS, FA, and SSS continuously enhanced the polymerization reaction efficiency, generated massive gel products, and considerably reduced the number of cracks and pores. Consequently, both the microstructure and compressive strength were continuously improved.

## Figures and Tables

**Table 1:** Chemical composition of Portland cement %.

Raw Material	SiO_2_	Al_2_O_3_	Fe_2_O_3_	CaO	MgO	Na_2_O	K_2_O	SO_3_	Others	Loss
FGDG	3.24	1.57	0.72	34.42	1.16	0.11	0.26	38.21	0.74	19.78
GBFS	34.65	20.72	0.55	34.23	5.21	1.47	0.65	-	1.26	0.79
FA	53.89	31.24	5.32	2.18	1.17	0.87	0.22	-	1.77	1.85

**Table 2:** Mix proportion of specimens/g.

	FGDG	GBFS	FA	SSS	Sodium Hydroxide	Water	Sand	Liquid-Solid Ratio
L-1	450/300	0/0	0/0	0/0	20/13	225/90	1350/0	0.5/0.3
L-2	405/270	45/30	0/0	0/0	20/13	225/90	1350/0	0.5/0.3
L-3	360/240	90/60	0/0	0/0	20/13	225/90	1350/0	0.5/0.3
L-4	315/210	135/90	0/0	0/0	20/13	225/90	1350/0	0.5/0.3
L-5	270/180	180/120	0/0	0/0	20/13	225/90	1350/0	0.5/0.3
L-6	225/150	225/150	0/0	0/0	20/13	225/90	1350/0	0.5/0.3
L-7	315/210	90/60	45/30	0/0	20/13	225/90	1350/0	0.5/0.3
L-8	270/180	90/60	90/60	0/0	20/13	225/90	1350/0	0.5/0.3
L-9	225/150	90/60	135/90	0/0	20/13	225/90	1350/0	0.5/0.3
L-10	180/120	90/60	180/120	0/0	20/13	225/90	1350/0	0.5/0.3
L-11	135/90	90/60	225/150	0/0	20/13	225/90	1350/0	0.5/0.3
L-12	225/150	90/60	135/90	20/13	20/13	225/90	1350/0	0.5/0.3
L-13	225/150	90/60	135/90	40/26	20/13	225/90	1350/0	0.5/0.3
L-14	225/150	90/60	135/90	60/39	20/13	225/90	1350/0	0.5/0.3
L-15	225/150	90/60	135/90	80/52	20/13	225/90	1350/0	0.5/0.3

Note: The concentration of sodium hydroxide solution is 2.22 mol/L in mortar and 3.61 mol/L in paste.

**Table 3:** Compressive Strength Analysis Results MPa.

	3d	28d	60d		3d	28d	60d
L-1	3.9	6.1	7.3	L-9	8	14.7	18.5
L-2	6	10.2	12.1	L-10	7.2	13.3	16.9
L-3	7	11.9	14	L-11	6.3	11.3	14.2
L-4	7.2	12.4	14.5	L-12	9.1	16.9	21.4
L-5	7.3	12.6	14.7	L-13	9	17.1	21.6
L-6	7.4	12.8	14.9	L-14	8.6	16.3	20.8
L-7	7.4	13.1	16	L-15	8.1	15.2	19.1
L-8	7.8	14.1	17.6				
